# Draft Genome Sequence of a New Delhi Metallo-β-Lactamase (NDM-1)-Producing Providencia stuartii Strain Isolated in Lima, Peru

**DOI:** 10.1128/MRA.00788-20

**Published:** 2020-09-24

**Authors:** Lizet Lezameta, Diego Cuicapuza, Alejandra Dávila-Barclay, Susan Torres, Guillermo Salvatierra, Pablo Tsukayama, Jesús Tamariz

**Affiliations:** a Laboratorio de Resistencia Antimicrobiana e Inmunopatología, Universidad Peruana Cayetano Heredia, Lima, Peru; b Laboratorio de Genómica Microbiana, Facultad de Ciencias y Filosofía, Universidad Peruana Cayetano Heredia, Lima, Peru; c Instituto de Medicina Tropical Alexander von Humboldt, Universidad Peruana Cayetano Heredia, Lima, Peru; d Wellcome Sanger Institute, Hinxton, United Kingdom; University of Rochester School of Medicine and Dentistry

## Abstract

Providencia stuartii is an opportunistic pathogen of the *Enterobacteriales* order. Here, we report the 4,594,658-bp draft genome sequence of a New Delhi metallo-β-lactamase (NDM-1)-producing Providencia stuartii strain that was isolated from an emergency patient in a private clinic in Lima, Peru.

## ANNOUNCEMENT

Providencia stuartii is a motile Gram-negative bacterium of the *Morganellaceae* family that is commonly found in the human gut microbiota (1). It is an opportunistic pathogen in patients with urinary catheters and a cause of urinary tract infections (UTIs) (2). It displays intrinsic resistance to polymyxins, nitrofurans, sulfamethoxazole, tetracyclines, fluoroquinolones, and aminoglycosides (3). Outbreaks of carbapenem-resistant P. stuartii have been reported in intensive care units in Brazil (4), Greece (5), and Saudi Arabia (6). Here, we present the draft genome sequence of the first New Delhi metallo-β-lactamase (NDM-1)-positive P. stuartii isolate reported in Peru.

Isolate PS901 was obtained from a urine sample from a 76-year-old patient in the emergency service of a private clinic in Lima, Peru. The patient was diagnosed with a UTI, unspecified encephalopathy, and septicemia. Ethical approval (approval number 508-2019/DM-CCPJ) was obtained from the institutional review board at the Japanese-Peruvian Centennial Clinic (Lima, Peru). A standard urine culture was performed in blood agar and CHROMagar orientation medium (Becton, Dickinson, Heidelberg, Germany). The culture was positive after 24 h with >100,000 CFU/ml and identified as Providencia stuartii with the Vitek 2 Compact instrument (bioMérieux, France). Genomic DNA of the isolate was extracted using the column-based GeneJET genomic DNA purification kit (Thermo Fisher Scientific, Waltham, MA, USA) following the manufacturer's instructions. Sequencing libraries were prepared using the Nextera XT DNA library preparation kit (Illumina, Inc., San Diego, CA, USA) and sequenced on an Illumina MiSeq instrument using a v2 reagent kit, generating 1,205,030 paired-end 250-bp reads.

The European UseGalaxy server (http://usegalaxy.eu) (7) was used to conduct all data analyses. Raw sequencing reads were quality checked, trimmed, and *de novo* assembled using FastQC v0.72 (8), Trimmomatic v0.36 (9), and SPAdes v3.12.0 (10), respectively, resulting in a draft genome of 132 contigs larger than 200 bp (*N*_50_, 193,853 bp), a total length of 4,608,029 bp with a mean coverage of 65-fold and a GC content of 41.68%. The NCBI Prokaryotic Genome Annotation Pipeline (PGAP) v4.11 (11) predicted 4,170 protein-coding genes, 75 tRNA genes, and 36 rRNA genes. Default parameters were used for all software tools unless otherwise noted. ABRicate v0.9.8 (12) was used to identify antibiotic resistance and virulence genes using the NCBI Bacterial Antimicrobial Resistance Reference Gene Database (13) and the Virulence Factor Database (VFDB) (14). Fourteen antibiotic resistance genes were identified using a sequence similarity threshold of 90%, including genes producing resistance to aminoglycosides [*rmtG*, *aac(6')-Ib-cr*, and *aac(2')-Ia*], sulfonamides (*sul1* and *sul2*), fluoroquinolones (*qnrVC1*), trimethoprim (*dfrA6*), β-lactams (*bla*_OXA-1_ and *bla*_TEM-135_), tetracyclines (*tetB*), phenicols (*catA2*, *catA3*, and *catB3*), and rifampin (*arr-3*). We identified the *cheY* (chemotaxis regulatory protein), *flhC* (flagellar biosynthesis transcription activator), *hcp-2* (type VI secretion system substrate), *kdsA* (2-dehydro-3-deoxyphosphooctonate aldolase), and *gmhA* (phosphoheptose isomerase, previously designated *lpcA*) virulence genes. The *bla*_NDM-1_ gene was identified in an 18,480-bp contig, followed by *ble*_MBL_ (a gene producing resistance to bleomycin), coinciding with previous reports (15, 16). A query against the PlasmidFinder database (17) identified plasmid replicon IncA/C2, which is commonly associated with *bla*_NDM-1_ (18).

This draft genome of P. stuartii reveals the dissemination of the *bla*_NDM_ gene in a nontraditional bacterial host and highlights the potential threat to public health of carbapenem resistance in Latin America.

### Data availability.

The draft genome assembly of Providencia stuartii PS901 has been submitted to NCBI under BioProject number PRJNA627256, and the raw reads have been submitted to the SRA under accession number SRR11611425.
